# Sporadic Hemiplegic Migraine with Seizures and Transient MRI Abnormalities

**DOI:** 10.1155/2011/258372

**Published:** 2011-09-25

**Authors:** Harsha Bhatia, Fawzi Babtain

**Affiliations:** ^1^Department of Neurology, Aseer Central Hospital, Abha, Saudi Arabia; ^2^Department of Neurology, King Khalid University, Abha, Saudi Arabia

## Abstract

Hemiplegic migraines are characterised by attacks of migraine with aura accompanied by transient motor weakness. There are both familial and sporadic subtypes, which are now recognised as separate entities by the International Classification of Headache Disorders, edition II (ICHD-II). Sporadic hemiplegic migraine is a rare variant of migraine, We report a case of sporadic hemiplegic migraine and seizures with MRI features suggestive of cortical hyper intensity and edema on T2 and FLAIR images with no restriction pattern on diffusion and these changes completely resolving over time, suggesting that these changes are due prolonged neuronal depolarization and not of ischemic origin.

## 1. Introduction

Migraine affects about 12% of the population in Western countries. Familial hemiplegic migraine (FHM) is the only variety of migraine characterized by an autosomal dominant pattern of inheritance and transient hemiparesis followed by migraine headache [1]. Some sporadic cases of hemiplegic migraine with cerebellar signs have also been reported [2]. Sporadic hemiplegic migraine (SHM) has clinical symptoms identical to FHM and distinct from migraine with aura. We report a case of sporadic hemiplegic migraine who had prolonged right hemiparesis with aphasia and seizures focal characteristic MRI findings suggestive of diffuse cortical hyperintensity on T2 and FLAIR images, which resolved completely over a period of time. This is a rare case report of association of sporadic hemiplegic migraine with seizures and such MRI findings.

## 2. Case Report

A 24-year-old female was initially presented in our ER with a history of left-sided throbbing headaches associated with blurring of vision followed later by development of right-sided hemiparesis and aphasia and later started having right focal motor seizures. 

The past medical history dated about 12 years back when she had her first episode of headache with right-sided weakness and had been having such episodes infrequently and was alternating with right and left sides, lasting for few hours and then reverting back to normalcy preceded always with headache unilateral, throbbing in character associated with nausea and sometimes with photophobia. The patient has been admitted and investigated; her CT scan head, MRI brain, angiogram were normal, CSF lactate was also normal along with serum lactate, other biochemical parameters were normal, and the connective tissue disease was ruled out by relevant investigations. Since there was no family history, she was diagnosed as having sporadic hemiplegic migraine. The patient was started on flunarizine (Sibelium 10 mg). Later she started having right focal seizures preceded with headache and right hemiparesis, now the headache localizing always to left side, her medication was changed to Topiramate which was gradually escalated, in spite of that she was having such attacks once or twice a month, this was the last attack when she came to our hospital. 

Examination on admission revealed young pleasant-looking female who was consciously cooperative and had motor aphasia with right hemiparesis with normal sensation and fundal examination. Other systemic examination was normal. The patient was investigated CBC Hb 12.7 gm. 

WBC 6.0, PLT 223, and metabolic biochemical panel were normal; thyroid function test normal, ANA, anti-DNA negative, ACLA, APL, Beta 2 glycoprotein negative, and Serum lactate as well as CSF lactate was normal. EEG showed no hemispheric dysfunction.

## 3. Imaging Findings

**Figure 1:**
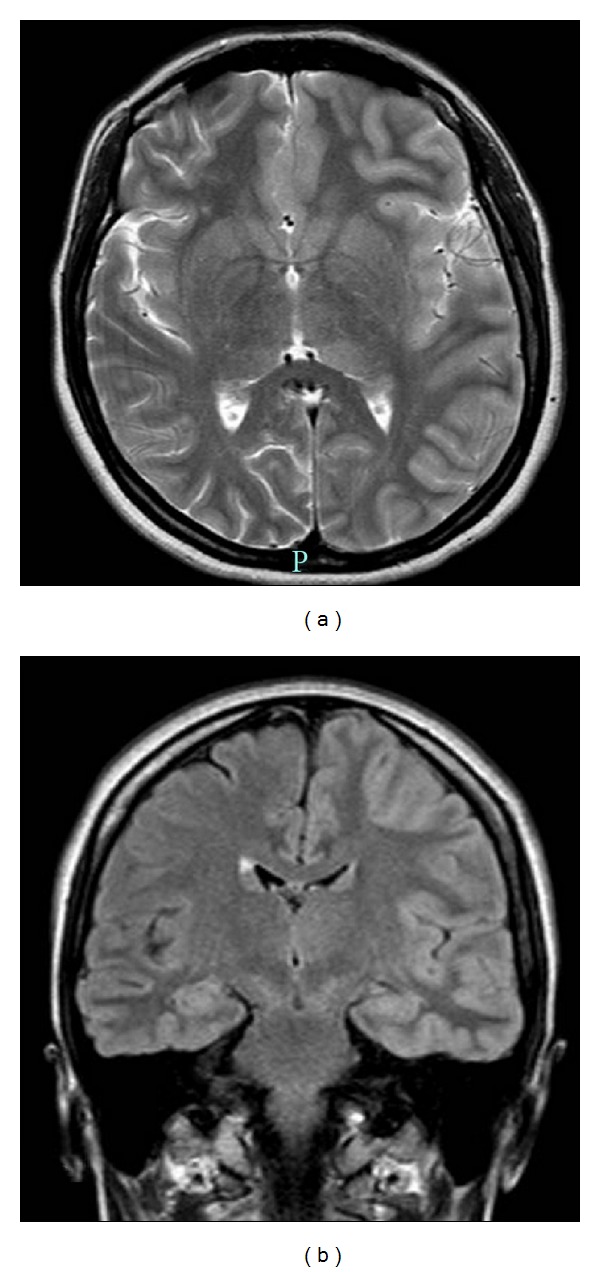
Follow-up MRI of the patient. Coronal fast spin-echo T2-weighted MR images (a) and Flair images (b) through the level of the Sylvian fissures show normal MRI study with disappearance of the previously noted cortical swelling and hyperintensity of the left cerebral hemisphere.

MRI was done for the patient at the time of presentation (Figure 1). T2-weighted and FLAIR images showed diffuse cortical swelling and mild cortical hyperintensity of the left hemisphere. These findings were most likely caused by cortical edema. Diffusion-weighted images were normal enabling us to rule out an acute brain infarction. T1-weighted images and MR angiograms were normal. Postcontrast study showed no abnormal enhancement. There was no tumor, obvious lesion, or sign of infection, such as abscess or encephalitis, on MR images.

**Figure 2:**
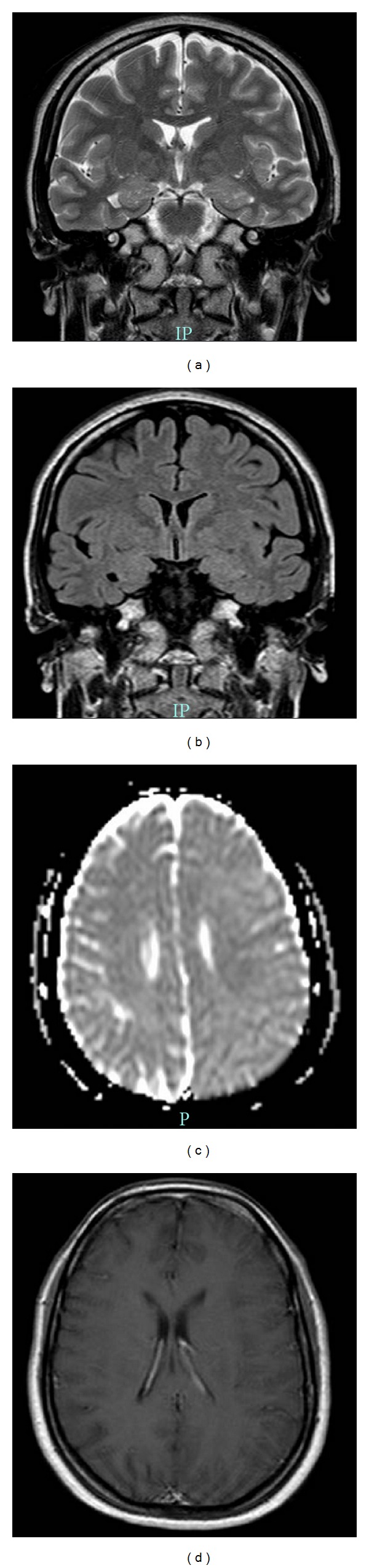
MRI of the patient at the time of presentation. (a) Transverse fast spin-echo T2-weighted MR images through the level of the sylvian fissures show diffuse cortical swelling and mild cortical hyperintensity of the left cerebral hemisphere. These findings are most likely caused by cortical edema. (b) Coronal diffusion-weighted MR image through the level of the basal ganglia shows diffuse high signal of the cortex of the left cerebral hemisphere. (c) Diffusion-weighted images show no evidence of water restriction in the left hemisphere. (d) Postcontrast study shows that the area does not enhance on T1-weighted images after contrast medium.

The patient was continued on topiramate, and the dose was escalated up to 100 mg bd, and she improved. After six months, symptoms had fully recovered, and MR imaging was repeated and showed normal findings. The previously noted MRI abnormalities on T2-weighted images, FLAIR images, and diffusion-weighted images disappeared (Figure 2).

## 4. Discussion

Hemiplegic migraine was initially described in 1910 as a type of migraine consisting of recurrent headache associated with transient hemiparesis. Familial hemiplegic migraine (FHM) is a rare autosomal dominant form of migraine with aura in which some degree of hemiparesis is present during attacks. In FHM, the aura typically lasts longer than in migraine with aura and usually comprises visual, sensory, aphasic, and motor symptoms [3]. However, patients with similar clinical symptoms but without other affected family members have been reported, and the term sporadic hemiplegic migraine has been used. Based on the literature, two subforms of FHM families exist—pure FHM in 80% and FHM families with cerebellar symptoms in 20% [4]. Mutations in the gene CACNA1A, which encodes a neuronal calcium channel, are present in 50% of families with hemiplegic migraine including those with cerebellar signs [1]. Eight mutations in CACNA1A have been identified in 18 families affected by hemiplegic migraine and two patients with SHM [5]. Genetic studies have revealed de novo mutations or incomplete penetrance of CACNA1A in patients with SHM [1, 2], thereby suggesting that SHM may be part of the spectrum of FHM syndromes. SHM has clinical symptoms identical to FHM. In SHM, presence of motor aura symptoms is essential. Other common aura symptoms are sensory (98%), visual (91%), and some form of aphasia (81%). Up to 72% of patients with SHM have concurrence of basilar migraine. Headache almost always occurs in close temporal relation to the aura, and SHM has an age of onset younger than 45 years [6]. The most differential diagnoses of SHM typically includes epilepsy (postictal weakness following seizure, or Todd's phenomenon), transient ischemic attack or stroke, metabolic abnormalities associated with focal deficits (hypercapnia, hypoglycemia, hyponatremia, hypocalcemia, hepatic failure, and renal failure), meningitis or encephalitis, carotid dissection, antiphospholipid antibody syndrome, SLE, and ornithine transcarbamylase deficiency.

Inherited disorders associated with migraine headache that may include hemiparesis include cerebral autosomal dominant arteriopathy with subcortical infarcts and leucoencephalopathy (CADASIL); mitochondrial myopathy, encephalopathy, lactic acidosis, and stroke-like episodes (MELAS); hereditary hemorrhagic telangiectasia (HHT); hereditary cerebral amyloid angiopathy; familial cerebral cavernous malformation; benign familial infantile convulsions [6].

The association of abnormal MRI findings and SHM is poorly understood due to the rarity of the entity and paucity of the literature. The data on imaging abnormalities described in sporadic hemiplegic migraine as well as familial hemiplegic migraine is sparse. The findings described in the literature consist of either restricted diffusion, normal or increased diffusion based on DWI and ADC values involving a hemisphere opposite to the side of deficit [5, 7, 8] associated with normal T2W and T1W images; angiography and perfusion studies have shown hyperperfusion [7]. However, in the present case, there was evident MRI abnormality which was in the form of presence of hyperintense areas on T2-weighted images and FLAIR images, in addition to no restricted diffusion pattern seen on DWI.

These changes could possibly suggest vasogenic edema or a metabolic change as evidenced by complete reversibility on followup, both clinically and radiologically. The demonstration of a fully reversible metabolic abnormality without vascular occlusion in the context of clinical migrainous-infarction indicates that the pathophysiology of this process is at the cellular level. Hemiplegic migraine is the most likely possibility. She had no family history. We therefore believe that she has sporadic hemiplegic migraine (SHM). Very similar MRI and magnetic resonance angiographic changes have been demonstrated in FHM. By extrapolation, the findings in our case suggest that the mechanism underlying SHM are similar to FHM. Beside this, our patient had seizures (partial) and were preceded by migraine; such focal lesion in MRI is possibility because of such seizures. The possibility that transient brain MRI abnormalities in a patient with migraine with aura followed by seizures may be due to migralepsy should be borne in mind to avoid misdiagnosis and potentially aggressive procedures [9].
